# Smart System with Artificial Intelligence for Sensory Gloves

**DOI:** 10.3390/s21051849

**Published:** 2021-03-06

**Authors:** Idoia Cerro, Iban Latasa, Claudio Guerra, Pedro Pagola, Blanca Bujanda, José Javier Astrain

**Affiliations:** 1 IED Electronics, Pol. Ind. Plazaola, E6, 31195 Berrioplano, Spain; icerro@iedelectronics.com (I.C.); ilatasa@iedelectronics.com (I.L.); 2 Department of Statistics, Computer Science and Mathematics, Public University of Navarre, 31006 Pamplona, Spain; pedro.pagola@unavarra.es (P.P.); blanca.bujanda@unavarra.es (B.B.); 3 Plant Pamplona SAS Autosystemtechnik, S.A., Faurecia, Polígono Industrial de Arazuri-Orcoyen, 31170 Arazuri, Spain; claudio.guerra@faurecia.com; 4 INAMAT2-Institute for Advanced Materials and Mathematics, Public University of Navarre, 31006 Pamplona, Spain; 5 Institute of Smart Cities, Public University of Navarre, 31006 Pamplona, Spain

**Keywords:** sensory gloves, machine learning, convolutional neural networks, smart sensing, automotive industry, industry 4.0, electronics

## Abstract

This paper presents a new sensory system based on advanced algorithms and machine learning techniques that provides sensory gloves with the ability to ensure real-time connection of all connectors in the cabling of a cockpit module. Besides a microphone, the sensory glove also includes a gyroscope and three accelerometers that provide valuable information to allow the selection of the appropriate signal time windows recorded by the microphone of the glove. These signal time windows are subsequently analyzed by a convolutional neural network, which indicates whether the connection of the components has been made correctly or not. The development of the system, its implementation in a production industry environment and the results obtained are analyzed.

## 1. Introduction

Within the demanding automotive sector, it is essential to have high quality standards and low defective product ratios (NOK—not OK) at the end of a production line (EOL—End of Line) if adequate levels of competitiveness are to be maintained. In order to comply with this, companies incorporate in their EOL a final validation and testing process through electrical checks (E-CHECK) that ensure that all functions and connections of a product are correct and that there are no defects.

The connection of the wiring is made by means of a click that generates a characteristic sound that the operator must detect, thus ensuring that the connection (clicking) has been made correctly. However, this process involves a high degree of dedication of factory personnel, which increases manufacturing costs. In addition, on many occasions, these solutions do not ensure a perfect clicking of the connectors, which, with the passage of time, end up disconnecting and deriving in complaints from clients and the need to repair the product, deriving in a very high cost.

The aim of this paper is to describe a new sensory system capable of carrying out the verification of the correct embedding in the cable connection that is done in many production lines, with success rates of almost 100%. The sensory system will be integrated into some sensory gloves to carry out the verification of the correct packaging in real time along the entire production line, without having to resort to the E-CHECK in the EOL. The system provides a high degree of flexibility, being able to operate in different environmental conditions within industrial environments increasing the reliability of the processes of checking the proper clicking connection. The system also aims to simplify the E-CHECK in EOL and balance the load along the different assembly stations of a cockpit module, reducing significatively the total time of assembly and testing of a cockpit module. The reduction of the number of NOK cockpits at the end of the line by means of early and autonomous detection (self-assessment) of the incorrect clicking connection along the whole assembly line will imply a reduction of the manufacturing costs of the assembly process of the cockpit module, thus offering a product at a more competitive price. Furthermore, it allows getting an integrated and highly configurable system to adapt to the needs of the operator and to digitize the production systems by incorporating 4.0 technologies.

## 2. Related Works

The use of smart gloves is not new at all. As described in [1], “hand movement data acquisition is used in many engineering applications”. The use of sensory gloves has been considered for many purposes, such as sign language recognition [2,3], hand posture monitoring [4,5], computer-generated (typically virtual reality or augmented vision) environments [6], tactile sensing [7,8,9,10], force-sensing for biomedical purposes [11,12,13], fitness exercises tracking [14], Sensing Finger Tapping in Piano Playing [15], teleoperation [16], rehabilitation [17,18,19] and many others. However, the use of convolutional neural networks (CNNs) to provide intelligence to these gloves is indeed a significant improvement in the recognition capacity of these devices.

A complete and interesting survey of force feedback is proposed in [20], while [21] classifies wearable haptic interfaces and presents a taxonomy of these interfaces. All this allows us to understand the contribution that these types of devices and techniques can make to their successful application in the problem of connection detection that concerns us.

Instrumented gloves may include different sensors as microphones, force sensors, proximity sensors, accelerometers (ACCs), gyroscopes, flexion (bend) sensors and many others. Furthermore, a natural feature of these systems is mobility, so they are wireless devices, with a limited computing capacity (often cloud- or edge-based computing systems) and with a limited energy autonomy determined by the batteries they are able to carry (without losing user’s ergonomic). Of course, there are numerous commercial proposals, as SensoGlove (http://www.sensoglove.com/) (accessed on 5 Marth 2021), ProGlove (https://www.proglove.com) (accessed on 5 Marth 2021), CyberGloveII (http://www.cyberglovesystems.com/cyberglove-ii) (accessed on 5 Marth 2021), VRfreeGloves (www.sensoryx.com) (accessed on 5 Marth 2021) and many others, which provide general purpose or specific oriented solutions for many kind of problems.

In our case, we propose the use of convolutional neural networks to detect the proper connection of equipment on the dashboard of vehicles using work gloves that include a microphone, a gyroscope and accelerometers. Due to the very nature of the vehicle manufacturing system involving wear and deterioration of gloves, they must be low-cost and, in addition, ergonomic, lightweight and sensitive to the touch. Then, a wireless sensor prototype has been developed, which integrates the components mentioned above, and communicates wirelessly, by means of Blueetooth Low Energy (BLE) with the automatic clicking recognition system.

The contribution lies in the very characteristics of the CNNs used. These CNNs offer high recognition rates, and therefore a considerable reduction of defective product ratios (NOKs); and on the other hand, can benefit from the use of the gloves in the production process, since a retraining of the system is proposed, taking advantage of the non-productive periods of the factory, as described below.

The rest of the paper is organized as follows: Section 3 is devoted to describing the system proposed and the industrial environment where it will work; Section 4 is devoted to explain the smart recognition system, including the convolutional neural system developed; Section 5 describes the implementation of the system; Section 6 describes and discusses the results obtained; Section 7 shows the conclusions, and finally, references end the paper.

## 3. System Description

This section describes the industrial environment in which the system will work. It also describes the characteristics of the signals to be measured, the architecture of the proposed system and the intelligence of the system.

### 3.1. Industrial Environment

**Figure 1:**
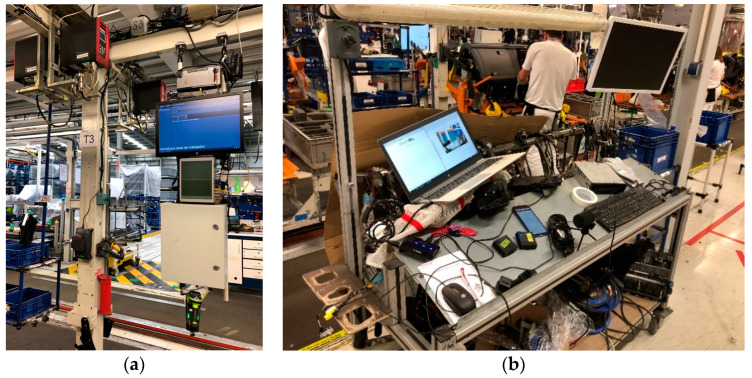
Assembly line segment (**a**) and experimental measuring and testing workstation (**b**).

The working environment of the application is usually located in a hostile environment as far as noise sources are concerned, reaching noise values of 90 dB on a stationary basis. As described in [22], lighting and noise levels affect to human productivity in the automotive assembly industry. Typically, workers performing similar tasks and especially noise sources, generally of an impulsive nature, such as pneumatic screwdrivers, hammers, metal and plastic tools, robots, forklifts, chains and gears, coexist near the workplace. Figure 1 shows the industrial environment where the system must work. There is a continuous movement of components, accompanied by the movement of elements through the assembly line, the movement of transport trucks, the operators’ own conversations, and many more sources of noise.

In order to characterize the work environment, almost 100 h of uninterrupted 24-hour-a-day work have been recorded at some locations were clicking occurs on the assembly line, using the same audio sensors that will be described later (microphones on the glove itself and on the outside). These recordings have included all the work shifts of that day. The purpose of recording this noise is not only to know the background noise of the environment, but also to have noise sources to synthetize negative samples for later use in the training of neural networks and to generate synthetic samples adding laboratory clicking signals.

### 3.2. Signals and Their Acquisition

**Figure 2:**
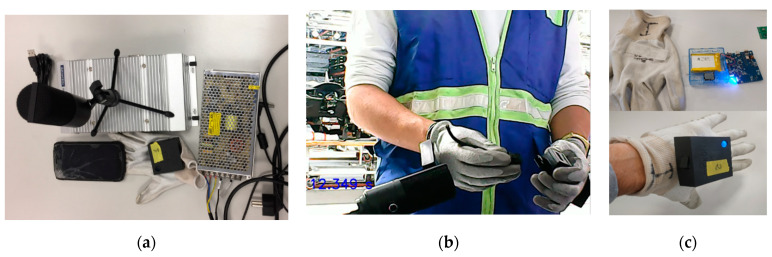
Measurement: (**a**) equipment used, (**b**) recording of the embedding process with an external microphone, (**c**) glove with the acquisition system.

For the recording of the embedding signals, as well as the operator’s movement, some sensors have been used, such as, microphones, accelerometers, gyroscopes and video cameras, all of them synchronized in time (see Figure 2a). These elements are described below, as well as the signals obtained. Figure 2b shows the recording of the embedding process. In this case with an external microphone. The operator is equipped with a wireless sensor kit (NRF6936, from Nordic Semiconductor) attached to the top of each hand, as depicted in Figure 2c. This kit includes a digital MEMS microphone (MP34DB02, from ST Microelectronics) that records audio signals with a sampling frequency of 8 kHz and 16bits resolution, an Inertial Measurement Unit–IMU-(MPU-9250 from Inven Sense Inc.) that includes an accelerometer and a gyroscope, both with 3 axes, that register simultaneously at a rate of 100 Hz, with a range of ±2 G and a resolution of 16 bits in the case of the accelerometer and ±250°of range and 16 bits of resolution in the case of the gyroscope. The registers of both kits are sent by means of a Bluetooth Low Energy (BLE) interface to a PC, where data collected is stored. At the same time, the sound is registered at the PC with a capacitive microphone (FIFINE K669B from FIFINE MICROPHONE) located 10 cm from the place where the embedding takes place. Sound is sampled at a frequency of 44,100 kHz with a 16 bits resolution. Simultaneously, a webcam (VF0260, Creative Labs Inc., Singapore) records the embedding process in order to help determine the exact instant when embedding occurs. Table 1 summarizes the features of all the described components.

**Figure 3:**
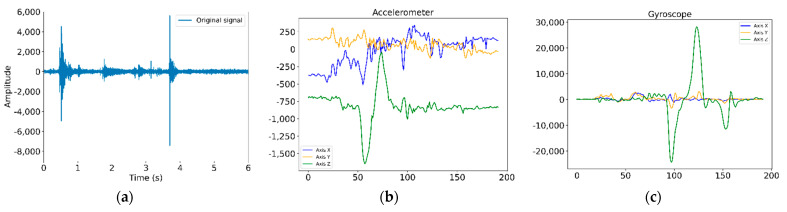
Signals: (**a**) acoustic signal obtained, (**b**) 3 axis accelerometer values obtained, (**c**) 3 axis gyroscope values obtained.

Figure 3 shows a sample of an audio signal acquired (a), the three axis (X, Y and Z) values obtained for the accelerometer (b) and the angular velocity measured by the gyroscope (c).

**Figure 4:**
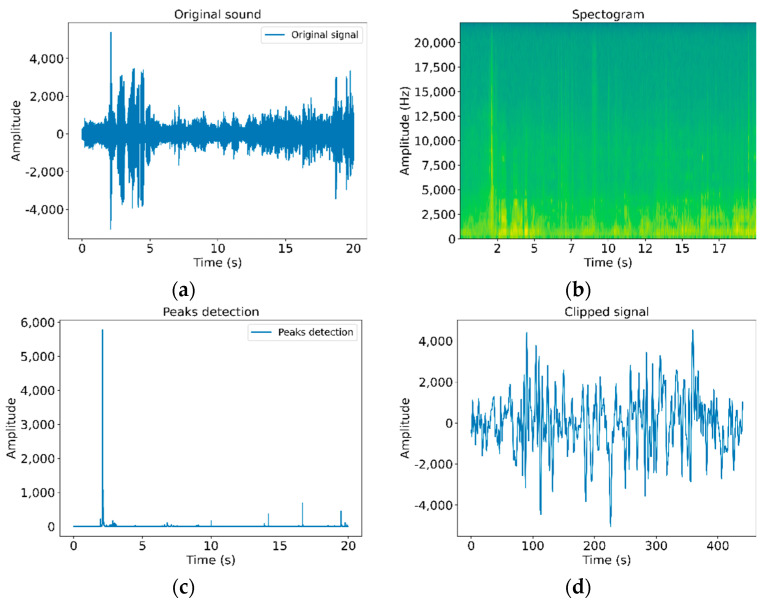
Signal processing: (**a**) original signal trimmed to 20 s, (**b**) spectrogram of the signal, (**c**) peaks detection, (**d**) signal window selected.

To detect the exact moment of when the clicking is performed, the signal acquisition process is followed by the calculation of the audio power spectrum of the signal (128 samples), as depicted in Figure 4b, considering an overlapping of 16 samples for each spectrum. Then, the sum of the spectral power in the range of 11 kHz is calculated, as shown in Figure 4c. The maximum relative values, higher than the detection threshold, are detected and a signal window is obtained. This window begins one millisecond before the trigger, as shown in Figure 4d. The resulting trimmed signal starts 44 samples before the detected peak (-frec/1000) and finishes 443 samples after the detected peak.

As expected, there is a wide range of devices to be interconnected, with many different morphologies and connectors, so that the signal characteristics change, and became in new challenges to determine correct clicks against external noise. For this reason, it is interesting to analyze different techniques to determine which of them are capable to detect the proper connection of the different devices in the dashboard of the vehicle. In this article, we have considered two devices: an electronic climate control (Climatronic) and a light dimmer (Dimmer).

**Figure 5:**
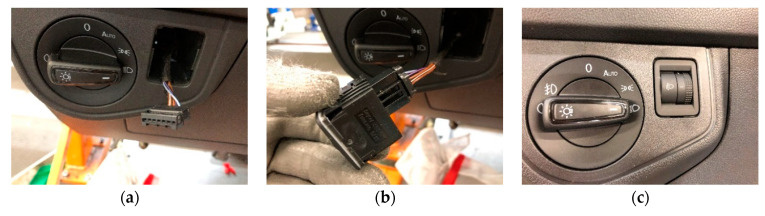
Dimmer installation: (**a**) connection wiring, (**b**) dimmer connection, (**c**) insertion of the dimmer in the dashboard.

**Figure 6:**
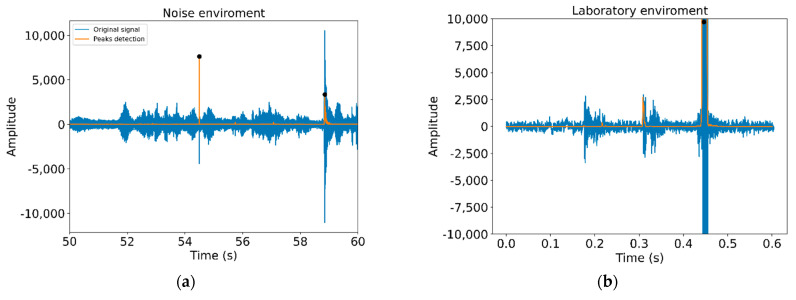
Noise level measured: (**a**) connection of a Dimmer in the production line (with noise), (**b**) connection of an RJ45 connector in a laboratory environment (without noise).

Figure 5 illustrates the installation of the Dimmer, while Figure 6 shows the observed difference in the noise levels obtained for two similar signals depending on the environment. In Figure 6, the image on the left shows the connection of a Dimmer in the production line (with noise), while the image on the right corresponds to the connection of an RJ45 connector in a laboratory environment (without noise). The Dimmer connection corresponds to a range of 10 seconds in which two clicks occur (marked with a black dot).

In order to acquire the signal samples, several measurement rounds were carried out. These captures include noise from the production line (Figure 1a) and samples of real connections performed on a specific experimental station very close to the line (Figure 1b). In the case of the actual assembly samples, correct and incorrect assemblies were made in order to have a complete set of samples to work with. Noise recording from the production line was used to build synthetic samples.

### 3.3. System Architecture

**Figure 7:**
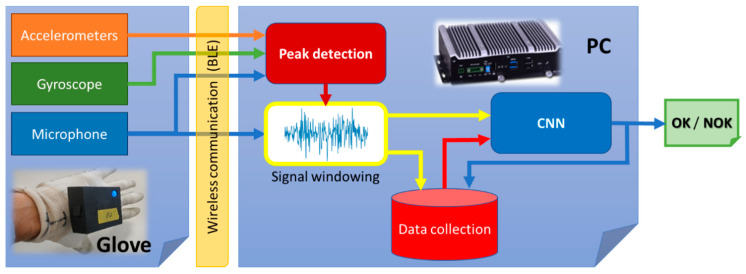
System architecture: the glove, which includes the accelerometers, gyroscope and microphone (**left**), the Bluetooth Low-Energy wireless communication interface (**middle**) and the personal computer (PC), which includes the peak detection subsystem, the windowing subsystem, the data collection database and the convolutional neural network (**right**).

The system consists of a glove that houses the sensors (microphone, gyroscope and accelerometers), which is placed in the dominant hand, and a small industrial microcomputer that recognizes the acquired signals and provides as output the conformity or non-conformity (OK/NOK) with the performed component connection made. Since the user’s mobility takes precedence over any other criteria, the glove and the microcomputer elements are interconnected through a Bluetooth Low Energy (BLE) link, which offers the best connectivity/energy-consumption ratio. Figure 7 shows the system architecture.

In both cases, glove and microcomputer, a reduced cost is sought. In the case of the glove, the capture device was secured initially by means of a Velcro fastener to facilitate the replacement of the glove with a new one when it is worn out. Once the viability of the prototype has been validated, work is currently being done on miniaturization and roughing up of the device to avoid any damage while guaranteeing ergonomics.

## 4. Smart Recognition System

This section is devoted to describe the recognition system, paying special attention to the description of the techniques used and the problems observed. The final implementation of the system will be described in the following section.

### 4.1. Accelerometer-Based Recognition

The use of accelerometers allows detecting the time windows to be sampled and clipped and then sending them to the convolutional network. Without the help of accelerometers, it would not be possible to identify the samples to be recognized and the process would be much more costly in terms of time and computation.

In order to automate as much as possible the identification and selection of the time windows of the signal (windowing) to be provided to the network, we try to identify the waveform of the accelerometers, as well as the peaks of the signal when a click (connection) is performed, by means of mathematical functional approximation. The identification of the movement made by the hand of the operator allows the system to identify the phase of the assembly process in which she/he is and thus to search adequately for the moments of connection of the connectors. Being able to approximate certain wave functions by means of mathematical functions makes it possible to significantly speed up the identification of the signal’s time windows, thus reducing the system’s computation time without having to over dimension the input matrix (signal) and without requiring continuous processing of the signals by the convolutional network.

The mathematical modelling of signals from sensors is generally complex due to the mathematically chaotic behavior of the sensors and to the great diversity of situations that can occur in the same movement. Mathematical processors based on computational algebra (such as Wolfram Mathematica (Wolfram Mathematica, https://www.wolfram.com/mathematica/) (accessed on 3 March 2021), Maxima Maxima, (https://maxima.sourceforge.io/index.html) (accessed on 3 March 2021) or Maple (Maple, https://www.maplesoft.com/) (accessed on 3 March 2021)) are not able, most of the time, to identify the behavior of such signals and connect it with analytical expressions of functions that allow proper manipulation.

**Figure 8:**
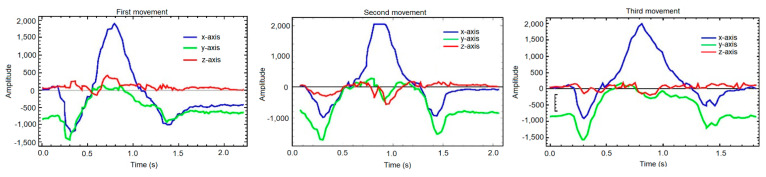
Three samples of the same circular wrist turn movement, and their variation over time in the three axes.

However, on many occasions it is possible to predict the behavior that a signal will have by carrying out specific studies, as we will describe below. Figure 8 shows, after filtering, transferring and scaling them properly, three samples where the operator has performed a circular twist of the wrist. As can be observed, although the different signals share a certain similarity in the waveform, the values do not match. That is, we can look for a pattern of movement that can be mathematically modeled with a function, but we must be aware that an imperfect matching will be required.

**Figure 9:**
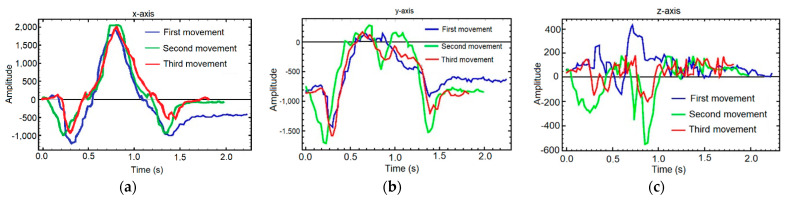
Samples overlapped by axis: (**a**) *X*-axis, (**b**) *Y*-axis, (**c**) *Z*-axis. Each graph shows the comparison between the first movement (blue), the second (green) and the third (red).

In order to be able to analyze each of the movements in the best possible way, they are compared by axis as depicted in Figure 9. Visually there is a clear difference between the three axes. In the X and Y axes there are big differences in the variation of the slopes of the curve, with a similar behavior in the three samples. However, in the Z axis the amplitude of the movement is smaller and it does not follow a clear structure. Mathematically, the absolute maxima and minima of the three graphs can be calculated and compared between them.

- In the first movement on the *X*-axis:
  Maximum=(0.79, 1933), minimum=(0.31, 1220)

The equation of the line connecting these points is y+1220=6568.89(x−0.314). The slope of this line is 6568.89.

- In the first movement on the *Y*-axis:
  Maximum=(0.735, 2048), minimum=(0.22, −984)

The equation of the line connecting these points is y+984=5937.63(x−0.224). The slope of this line is 5937.63.

- In the first movement on the *Z*-axis:
  Maximum=(0.81, 2009), minimum=(0.30, −925)

The equation of the line connecting these points is y+ 925=5749.74(x−0.3). The slope of this line is 5749.74.

**Figure 10:**
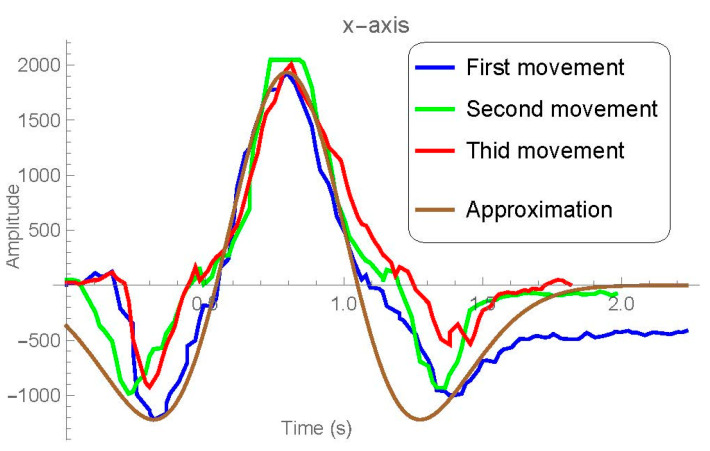
*X*-axis variation of the three motion cases and the approximation obtained (in brown).

In this comparison, the absolute highs and lows and the slopes are in the same range of variation (scaling may slightly influence positioning). Taking these references (as well as other more punctual and complicated ones that we did not indicate) it can be determined that the behavior in this axis is predictable and structured. In order to get an accurate approximation, we look for an analytic function f(x) that adjusts the data as best as possible. Figure 10 shows this approximation. Taking into account the previous data graphics, we can observe:

1. Variation must be insignificant at the beginning and the end of the movement, that is,$\underset{x\to \pm \infty }{\lim}f\left(x\right)=0$.
2. It’s clear (see Figure 10) that there are two time moments where the movement is minimum and other one where the movement has its maximum value, that is, f(x) must have two minimums $x=a_{1}\;$and $x=a_{3}$, and one maximum $x=a_{2}$ between them.

With these preliminaries, we choose $f\left(x\right)=K_{1}e^{-b{\left(x-a_{2}\right)}^{2}}p\left(x\right)$, with${\;K}_{1}\in R$, b>0 and p(x) a function smaller than $e^{-b{\left(x-a_{2}\right)}^{2}}$ when x is large, which mathematically means $p\left(x\right)=o\;\left(e^{-b{\left(x-a_{2}\right)}^{2}}\right)$ when |x|→∞. In this way, the simplest functions verifying this characteristic are the polynomials and that is what we will try to find. Now, we consider the derivative of f(x):

$$f^{\prime }\left(x\right)=K_{1}e^{-b{\left(x-a_{2}\right)}^{2}}\left(\;p^{\prime }\left(x\right)-2b\;\left(x-a_{2}\right)\;p\left(x\right)\right).$$

And, in order to verify condition 2, we impose that this derivative vanishes three times at $x=a_{1},\;a_{2\;}$and $a_{3}$

(1) $$p^{\prime }\left(x\right)-2b\;\left(x-a_{2}\right)\;p\left(x\right)=\left(x-a_{1}\right)\left(x-a_{2}\right)\left(x-a_{3}\right)$$

We also require $K_{1}>0$ with the end that $K_{1}\left(x-a_{1}\right)\left(x-a_{2}\right)\left(x-a_{3}\right)<0\;(\;f^{\prime }\left(x\right)<0\;)\;$and then f(x) decreases in $\left(-\infty ,\;a_{1}\right)\cup \left(a_{2}\;,\;a_{3}\right)$ and $K_{1}\;\left(x-a_{1}\right)\left(x-a_{2}\right)\left(x-a_{3}\right)\;>\;0\;(\;f^{\prime }\left(x\right)>0)\;$and then f(x) increases in$\;\left(\;a_{1}\;,\;a_{2}\;\right)\cup \left(a_{3}\;,\;+\infty \right)$. In other words, condition 2 is satisfied. Solving the differential Equation (1) we get

$$p\left(x\right)=C_{1\;}e^{b{\left(x-a_{2}\right)}^{2}}-\frac{1+b\;\left(x-a_{1}\right)\left(x-a_{3}\right)}{2\;b^{2}}+\left(2a_{2}-a_{1}-a_{3}\right)\frac{\;e^{b{\left(x-a_{2}\right)}^{2}}\sqrt{\pi \;b}}{4\;b^{2}}Erf\left(\sqrt{\pi \;b}\left(a_{2}-x\right)\right)$$

With $C_{1}\in R$ and Erf(z) the Error Function [23], a well known special function in the field of applied mathematics. On the one hand, we choose $C_{1}=0$ and condition 1 is satisfied. On the other hand, from experiment data we can observe that both minimum $a_{1}$ and $a_{3}$ are located symmetrically with respect to the maximum $a_{2}$, that is, we can consider

$$2a_{2}\;-\;a_{1}-a_{3}\approx \;0$$

With this assumption, the desired polynomial can be write as$\;p\left(x\right)\approx -\frac{1+b\;\left(x-a_{1}\right)\left(x-a_{3}\right)}{2\;b^{2}}$ and our approximation function reads

$$f\left(x\right)\approx K\;e^{-b{\left(x-a_{2}\right)}^{2}}\left(1+b\;\left(x-a_{1}\right)\left(x-2a_{2}+a_{1}\right)\right)\;$$

where we are recalled$\;K=\frac{K_{1}}{-2b^{2}}<0$. To determine K and b we can use the known (by data base) value of f(x) at $x=a_{1}\;\left(minimum\right)\;$and $x=a_{2\;}\left(maximum\right)$:

(2) $$f\left(a_{2}\right)=K\left(1-b{\left(a_{2}-a_{1}\right)}^{2}\right)=M_{1}>0\;\to \;\;b=\frac{1}{\;{\left(a_{2}-a_{1}\right)}^{2}}\;\left(1-\frac{M_{1}}{K}\right)>0$$

(3) $$f\left(a_{1}\right)=Ke^{-b{\left(a_{1}-a_{2}\right)}^{2}}\;=m_{1}<0\;$$

Putting Equation (2) into Equation (3) we get $K\;e^{\frac{M_{1}}{K}\;-1}\;=m_{1}$ and solving this transcendent equation we obtain K and subsequently the value of b from Equation (2) in terms of $f\left(a_{1}\right)=m_{1}$ and $f\left(a_{2}\right)=M_{1}$. With this procedure, we have completely determined an expression for f(x), which approaches the movement in the X axis and whose result has been shown in Figure 10.

This development just corresponds to the *X*-axis of the aforementioned movement. For the Y- axis we have followed a similar method, but the maximum is wider and the graph is shifted downward. In the case of the *Z*-axis, it is observed that there is no pattern worthy of mathematical modeling. All graphics in this section, as well as the necessary numerical approximations, have been made with Wolfram Mathematica 12.2.

### 4.2. Data Sources

**Figure 11:**
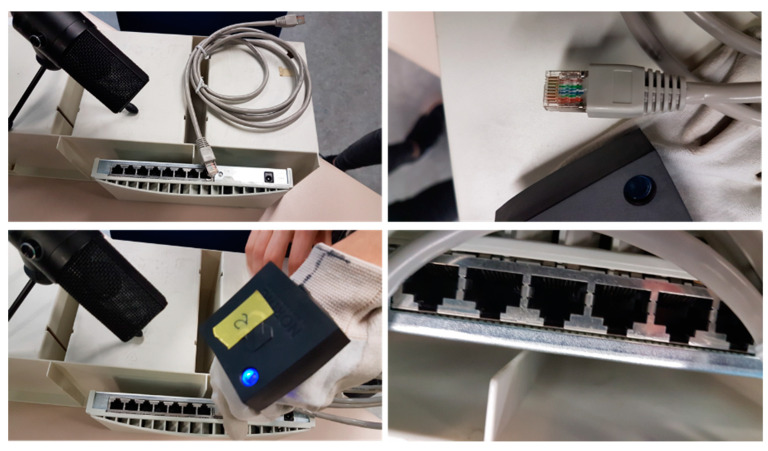
RJ-45 connection test bench: microphone, RJ-45 connector, sensory glove and network hub.

During the development process, we experienced additional difficulties when trying to acquire samples in the production plant including industrial noise. If it is usually already difficult and costly in time and effort to obtain permits to visit the plant to measure during the production process (obtaining a limited set of samples), the restrictions derived from the COVID19 pandemic caused a significant delay and forced us to build synthetic samples. For such reason, we carried out intense laboratory work to synthesize these samples, for which we used RJ-45 connectors and a network hub as shown in Figure 11.

In order to validate the proposed system, we proceeded to build an abundant set of synthetic samples from the connection of RJ-45 connectors. These samples included correct and incorrect clicking connections (incomplete connections, connections with broken connectors) in many different noise conditions. Environmental noise previously recorded in the production line was added to the laboratory samples, thus being able to build a set of samples with and without industrial noise. This allowed us to train the CNNs and validate their operation, and thereby validate the proposal as a step prior to training the CNNs with the samples acquired in the real production plant environment. Noise addition was performed by means of the Audacity (Audacity, https://www.audacityteam.org/ (accessed on 5 March 2021)) open-source audio editor.

**Figure 12:**
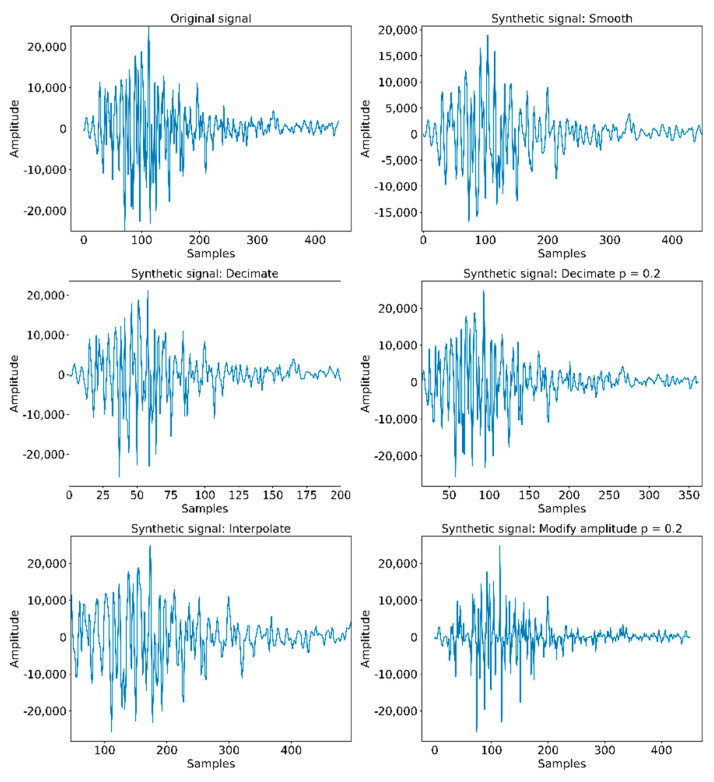
Example of synthetic signal generation.

Synthetic samples were created from the original ones through using different techniques such as:

1. Smoothing. The points of the signal are modified in such a way that those points that are higher than the adjacent ones (may be due to noise) are reduced, and those points that are lower than the adjacent ones are increased leading to a smoother signal. We obtain a sharper signal by means of a Savitzky–Golay filter, maintaining the original maximums and minimums.
2. Decimation. A new signal is generated, with a lower number of points than the original one. In our case, we set a constant decimating factor of 50%.
3. Deletion. Similar to signal decimation, but the elimination factor works under a user-imposed probability. A 30% in the case of the example depicted in Figure 12.
4. Interpolation. Method opposite to decimation, which constructs new data points within the range of the discrete set of known data points (probability of 50).
5. Modification of the amplitude. For each existing value, with a probability of 50%, its amplitude is modified a certain percentage delimited by the user. It can be expanded or reduced.

Table 2 shows the datasets used to train the CNNs. Ten sets of samples have been built. Two of them correspond to the RJ45 connectors obtained in the laboratory, while the remaining eight correspond to the Dimmer (four of them) and the Climatronic (the remaining four). The samples obtained at the laboratory correspond to the clicking of the connectors without ambient noise, while the synthetic laboratory samples include the environmental noise of the production line. On the other hand, we have the samples obtained at the production plant, at an assembly station close to the production line (see Figure 1b) in order to avoid any interference with the production process, but measuring under the same working conditions of the production line. Finally, the set of samples directly obtained on the assembly line during an actual production shift. All the datasets also include negative samples due to both ambient noise and bad connection because they actually occur during the process of sample acquisition and because they are of interest for CNN learning. Since the set of training samples is several thousand, which is relatively lower than the required values, we have increased the size of the training set using sample data augmentation from the samples obtained at the production line and also at the laboratory. The diversity of the data available for training models is then increased without having to collect new data.

The RJ45 datasets have just been used to train the network and check the system’s validity, measure the recognition capability and validate the network architecture described below. These laboratory samples have allowed us to properly calibrate the operating range of the glove sensors (microphone, gyroscope and accelerometers). Once proved the viability of the clicking recognition with the CNN, the network was trained with both the synthetic and real samples from the production plant.

### 4.3. Convolutional Neural Network (CNN)

The design and development of the convolutional neural network-based machine learning (CNN-based ML) has been carried out following the well-known criterion that 80% of the data bank goes to CNN training, while the remaining 20% is used in the final operation test. It should be noted that this is a supervised learning process that requires pre-tagging of samples. The tagging of the production line samples has been performed with the help of video cameras with capacity of temporary synchrony. With this, it has been possible to properly identify whether or not each detected event corresponds to a clicking event, and if this has been done, correctly.

**Figure 13:**
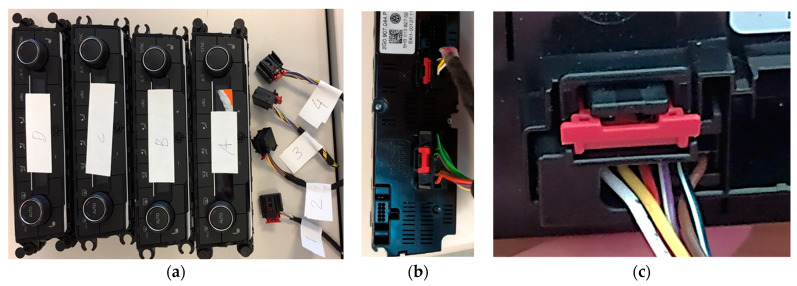
Climatronic: (**a**) devices and connectors, (**b**) back side, (**c**) connector.

A common convolutional neural network architecture has been built for both connectors (Dimmer—see Figure 5—and Climatronic—see Figure 13). Two different instances of the CNN have been developed, one for each device, following this architecture, and trained separately to acquire different weights. Each of the developed CNNs consists of three convolutional layers with max-pooling layers and four fully connected layers. Between them, a few layers have been added to avoid overfitting the model, a problem that happens when not having a large set of samples. Cross-entropy is used to estimate the loss function, as we are interested in penalizing erroneous predictions and obtaining good results with just two classes. Its optimization is performed using Adam’s algorithm [24] because this expression is optimal for image networks processing in cases like the one described above.

Table 3 summarizes the structure of the convolutional neural network designed. In the fully connected dense layers, an activation function must be added. This has been done using: (a) the ReLU layer, a rectified linear unit which allows to cancel the negative values from an activation map, increasing the nonlinear properties of the decision; and (b) the SoftMax function or normalized exponential function in the last dense layer, which finally returns the result. The SoftMax function is a generalization of the logistic function to multiple dimensions and it is typically used to normalize the output of the network and then located at the end of the network as the latest activation function. This causes a “compression” that gives very good results in combination with the cross entropy mentioned above.

**Figure 14:**
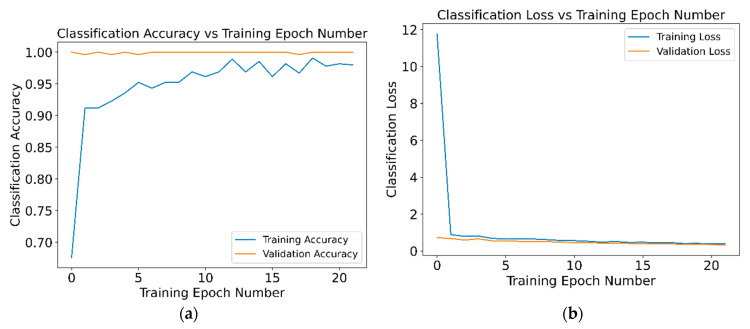
Dimmer Training Epoch Number: (**a**) Classification Accuracy, (**b**) Classification Loss.

In order to determine the number of training epochs to be used in the neural network, a study was carried out with the samples of the laboratory set of the Dimmer connector. Figure 14a shows the variation between the percentage of success with respect to the number of epochs, while Figure 14b shows the evolution of classification loss with the epoch number for the validation set. Results obtained for the Climatronic network are very similar.

The number of iterations in training was set to 10, after empirically verifying that the network behaves better when trained with 10 iterations as opposed to its training with 5 or 20 iterations. There is a 0.03 point increase in hit percentage and 0.1 point increase inaccuracy compared to 20 epochs. This shows that training with a higher number of epochs, and therefore more computationally expensive, does not offer a relevant benefit.

In order to validate the system and compare the different approaches used, five metrics have been considered and measured: accuracy, precision, recall, F1-score and specificity. The final confusion matrix is also extracted to analyze the network behavior. As it is well-known, accuracy is the ratio of correctly predicted observation to total instances, precision is the fraction of relevant instances among retrieved instances (true positive + false negative), recall is the fraction of retrieved relevant instances among all relevant instances (true positive + false positive), F1-score is the weighted average of precision and recall, and specificity is the fraction of negatives instances that are correctly identified among false positives.

### 4.4. Peaks Detection

So far, we have studied the prediction of true or false clicking samples, but in a real situation, these signals must also be found within the entire sampling period of the audio signal. That is, it is necessary to adjust the search window to locate the signal segment to be analyzed in order to validate the correct clicking. For this purpose, we have considered one minute of duration samples, in which ten correct connections are presented and a detection algorithm has been developed.

We start from the samples preprocessed by the same method discussed above. From there, we have defined basic rules that determine whether it is a connection or not. With the help of the mathematical approximations described above, we compare the signal values with the mathematical model that characterizes each searched peak and check if the observed signal is close enough to the searched model. For this purpose, we use a set of empirically determined thresholds and validate them with if-then-else conditions. A first set limit is the minimum amplitude that a peak must have to be considered valid. For example, for the connector described in Figure 14, it is a margin greater than 1000 amplitude, so all peaks detected below it are automatically discarded. From there, relative maximums have been calculated, since this is a feature that all successful connections meet. Subsequently, the nearest indexes to those points have been removed. This is because on certain connectors the peak is not clearly defined and has small local highs around its maximum value.

**Figure 15:**
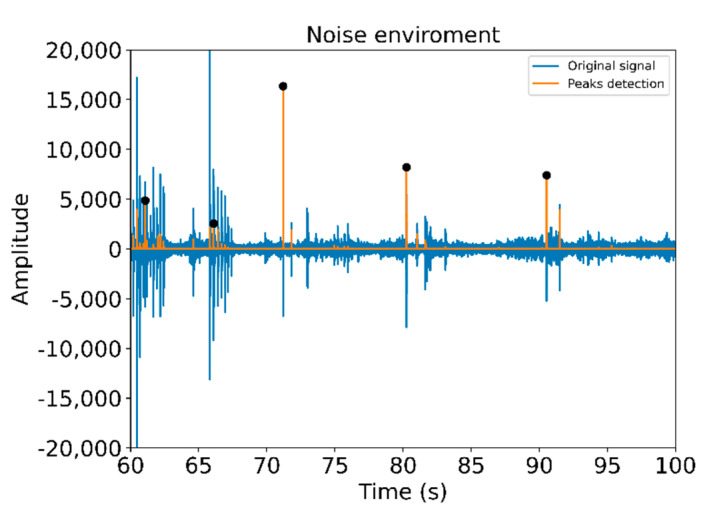
40 s audio sample and its corresponding peak detection.

To verify the accuracy and precision of this algorithm, all connections made have been manually tagged. In this way, precise time margins have been created in which a peak should exist. This allows to check whether the peak exist at that time, which would be a correct peak, or non-present, which would be an undetected peak. Furthermore, those detected outside the margins would be directly incorrect. Figure 15 shows a 40 s audio sample, in which we can observe five correct peaks. Other peaks are observed that do not meet the conditions of detection, and then they are not labeled as clicks.

## 5. System Implementation

As previously described, the system consists of two main elements: a wireless smart glove, responsible for signal acquisition and detection of a possible clicking connection, and a microcomputer, in charge of determining whether or not the connection has been made correctly. Both elements communicate with each other with a BLE wireless connection, and the computer interacts bi-directionally with the company’s logistics system.

The recognition system has been developed following 4 phases:

- Phase 1: Start of assembly. The logistics system indicates to the microcomputer the type of connector to be detected and, if necessary, its parameterization. In turn, the required information for detection is sent to the glove. The glove starts acquiring data from sensors. In anticipation of possible interruptions in the assembly process, a maximum period is set in which the signal will be captured. If this time limit is reached, an error is returned to the system and the recording made is discarded.
- Phase 2: Detection of possible clicking connection. The glove will sample continuously all analog signals (ACCs, gyroscope and audio), and will process in real time until a trigger of a possible clicking is detected. If this trigger occurs, the glove will send the clicking signal to the microcomputer. After that, the glove continues to sample uninterruptedly while waiting for more clicking.
- Phase 3: Clipping validity. The microcomputer will process the clicking, validating it or not, and notify the result of the event to the logistic system.
- Phase 4: End of registration. The device will end the registration when receiving the message from the computer of a successful ticketing, or by timeout, in that case a message will be sent to the microcomputer, indicating the latter to the logistic system that no clicking has been detected. In both cases, both the microcomputer and the glove wait to receive a new order from the logistics system.

Although the process may accumulate a certain delay, the result must be obtained in less than 5 s, which is the maximum delay accepted by the assembly line managers

The CNN implementation has been performed using two well-known open source libraries: Keras (Keras, https://keras.io/) (accessed on 5 March 2021), which acts as an API that allows the definition of the neural networks, and TensorFlow (TensorFlow, https://www.tensorflow.org/) (accessed on 5 March 2021), which is an automatic learning system (Deep Learning). Both libraries have been used with Python scripts over a couple of TTL TEKNO PRO computers equipped with 16 GB of DDR4 RAM memory, an Intel I5-8400 (2.11 GHz) processor with 4 kernels, 256 KB of L1 cache, 1 MB of L2 cache, and 6 MB of L3 cache memory. Both computers are devoted to the network training and run a Ubuntu 18.04 operating system.

## 6. System Validation: Experimental Results

The results of the final networks are shown below, both the original data set and the addiction of the synthetic samples.

In order to evaluate the contribution of each of the sample sets in the training of both networks (Dimmer and Climatronic), we have evaluated the results provided by both networks for each of the training sets. To do this, we have measured five key performance indicators (accuracy, precision, recall, F1 score, and specificity) for each CNN by validating with real samples obtained from the assembly line that have not been previously used at the training process.

First of all, we train the CNN with the samples captured in the laboratory (first row at Table 4), and then we add the synthetic samples generated from this same set but including real noise recorded from the assembly line and re-train the CNN (second row). We then re-train the CNN with the samples captured at the plant (third row), add again the corresponding synthetic set (fifth line), and finally we train the CNN with the four sets of samples (fourth row). We then obtain five different CNNs for each of type of device (Climatronic and Dimmer).

Table 4 shows the datasets of samples obtained for both devices (Dimmer and Climatronic). The table includes, for each training subset, the number of true negatives (TN, false clickings that the system recognizes as not performed), false positives (FP, something that is not a clicking but the system recognizes it as), false negatives (FN, a clicking not recognized as by the system) and true positives (TP, true clicking recognized as by the system). We can distinguish among the samples correctly catalogued (TN and TP), the samples incorrectly catalogued (FN and FP), the samples catalogued as OK (TP + TF) and the samples catalogued as NOK (FP + FN).

It is important to note that the validation over the CCN is just performed with the samples captured at the production plant.

Table 5 and Table 6 summarize, respectively, the key performance indicators of both Dimmer and Climatronic CNNs. As it can be observed, the Climatronic CNN offers better results than the Dimmer CNN, although the differences are rather small. Analyzing by connectors, best results correspond to the network trained with real samples (laboratory + plant) in the case of Dimmer, and in the case of Climatronic, best results correspond to the network trained with real samples (laboratory + plant) and the network trained with the full set of samples (Laboratory + synthetic + plant).

## 7. Conclusions

It has been proved for both samples captured at the laboratory and for real samples from a production plant that it is feasible, given a quality audio record, to determine whether the connections (clickings) performed have been made correctly (OK) or not (NOK).

It is appropriate to use synthetic signal samples while not having a large data set, and thus accelerate the process of knowledge. However, the use of real samples allows to obtain better final recognition results.

Automating the process of recognizing the clicking connections improves the quality of the final product and reduces production costs. Therefore, the results obtained show the viability and convenience of the use of the proposed system.

As future works, we identify the miniaturization and ruggedization of the glove, and the re-training of the networks as more samples are acquired during the operation of the system itself.

## Figures and Tables

**Table 1:** Features of the sensing components.

Sensor	Location	Range	Resolution	Sampling Rate
MEMS microphone	Both hands	120 dBSPL SNR 62.6 dB	16 bits	8 kHz
Acelerometer	Both hands	±2 g	16 bits	100 Hz
Gyroscope	Both hands	±250°	16 bits	100 Hz
Non-capacitive microphone	10 cm	120 dBSPL SNR 78 dB	16 bits	44,100 Hz
Video	A 1 m		640 × 480 pixel	30 Hz

**Table 2:** Sample sets obtained for the convolutional network training.

Connector	Samples Obtained at the Laboratory	Synthetic Laboratory Samples	Samples Taken at the Production Plant	Synthetic Production Line Samples
RJ45	2295	1792	-	-
Climatronic	1659	10,951	357	6257
Dimmer	1021	10,601	1164	10,775

**Table 3:** Architecture of the convolutional neural networks designed.

Type	Input Size	Output Size	Kernel Size	Number of Parameters
Conv2D	161 × 165 × 1	161 × 165 × 32	3 × 3	320
Max_Pooling2D	161 × 165 × 32	80 × 82 × 32	2 × 2	0
Conv2D	80 × 82 × 32	78 × 80 × 32	3 × 3	9248
Max_Pooling2D	78 × 80 × 32	39 × 40 × 32	2 × 2	0
Conv2D	39 × 40 × 32	37 × 38 × 32	3 × 3	0
Max_Pooling2D	37 × 38 × 32	18 × 19 × 32	2 × 2	0
Flatten	18 × 19 × 32	10,944	-	0
Dense	10,944	64	-	700,480
Dropout	64	64	-	0
Dense	64	32	-	2080
Dropout	32	32	-	0
Dense	32	16	-	528
Dropout	16	16	-	0
Dense	16	2	-	34

**Table 4:** Results obtained for both devices (Dimmer and Climatronic) for each of the training sets.

	Dimmer						Climatronic					
Dataset for Training	TN	FP	FN	TP	NOK	OK	TN	FP	FN	TP	NOK	OK
Laboratory	516	49	12	587	565	599	172	8	1	177	180	178
Laboratory + synthetic	536	29	27	572	565	599	180	0	9	169	180	178
Laboratory + plant	558	7	8	591	565	599	179	1	0	178	180	178
Labor + synth + plant	549	16	9	590	565	599	180	0	1	177	180	178
Plant + synthetics	528	37	1	598	565	599	178	2	0	178	180	178

**Table 5:** Results of the Dimmer CNN.

Dataset Used for Training	Accuracy	Precision	Recall	F1	Specificity
Laboratory	0.9476	0.9230	0.9800	0.9506	0.9476
Laboratory + synthetic	0.9519	0.9517	0.9549	0.9533	0.9519
Laboratory + plant	**0.9871**	**0.9883**	0.9866	**0.9875**	**0.9871**
Laboratory + synthetic + plant	0.9785	0.9736	0.9850	0.9793	0.9785
Plant + synthetic	0.9674	0.9417	**0.9983**	0.9692	0.9674

**Table 6:** Results of the Climatronic CNN.

Dataset Used for Training	Accuracy	Precision	Recall	F1	Specificity
Laboratory	0.9749	0.9568	0.9944	0.9752	0.9749
Laboratory + synthetic	0.9749	**1.0000**	0.9494	0.9741	0.9749
Laboratory + plant	**0.9972**	0.9944	**1.0000**	**0.9972**	**0.9972**
Laboratory + synthetic + plant	**0.9972**	**1.0000**	0.9944	**0.9972**	**0.9972**
Plant + synthetic	0.9944	0.9889	**1.0000**	0.9944	0.9944

## Data Availability

Data supporting reported results are owned by SAS Autosystemtechnik, S.A., Faurecia.
